# Pathological implications of metabolic reprogramming and its therapeutic potential in medulloblastoma

**DOI:** 10.3389/fcell.2022.1007641

**Published:** 2022-10-19

**Authors:** Veronica Marabitti, Manuela Giansanti, Francesca De Mitri, Francesca Gatto, Angela Mastronuzzi, Francesca Nazio

**Affiliations:** ^1^ Department of Hematology/Oncology and Cell and Gene Therapy, Bambino Gesù Children’s Hospital, IRCCS, Rome, Italy; ^2^ Department of Laboratory Medicine, Karolinska Institutet, Stockholm, Sweden

**Keywords:** OXPHOS (oxidative phosphorylation), metabolism, warburg effect, glutamine/glutamate (GABA) cycle, ROS

## Abstract

Tumor-specific alterations in metabolism have been recognized to sustain the production of ATP and macromolecules needed for cell growth, division and survival in many cancer types. However, metabolic heterogeneity poses a challenge for the establishment of effective anticancer therapies that exploit metabolic vulnerabilities. Medulloblastoma (MB) is one of the most heterogeneous malignant pediatric brain tumors, divided into four molecular subgroups (Wingless, Sonic Hedgehog, Group 3 and Group 4). Recent progresses in genomics, single-cell sequencing, and novel tumor models have updated the classification and stratification of MB, highlighting the complex intratumoral cellular diversity of this cancer. In this review, we emphasize the mechanisms through which MB cells rewire their metabolism and energy production networks to support and empower rapid growth, survival under stressful conditions, invasion, metastasis, and resistance to therapy. Additionally, we discuss the potential clinical benefits of currently available drugs that could target energy metabolism to suppress MB progression and increase the efficacy of the current MB therapies.

## Introduction

Medulloblastoma (MB) is a malignant brain tumor arising mainly in childhood, including four molecular subgroups–Wingless (WNT), Sonic Hedgehog (SHH), Group 3 and Group 4– that have been defined through transcriptome, methylome and genome profiling analyses (Northcott et al., 2017). MB generally arises in the posterior fossa and it is thought to initiate from an unusual neural stem/progenitor cell during early brain development. In one-third of patients, tumor cells spread to the leptomeninges via the cerebrospinal fluid, resulting in the formation of metastasis. The current standard of care includes maximal safe resection, radiotherapy and chemotherapy, according to patients’ stratification; patients frequently fail first-line therapy and are still often incurable (Packer et al., 2006; Jakacki et al., 2012; Veneroni et al., 2017; Juraschka and Taylor, 2019). Intertumoral heterogeneity in MB has long been recognized from a histopathological and a molecular perspective (Cavalli et al., 2017; Morrissy et al., 2017; Juraschka and Taylor, 2019) and very recently from the composition of the tumor immune microenvironment (Bockmayr et al., 2018; Grabovska et al., 2020). Lately, single-cell sequencing studies have also revealed the presence of a profound clonal and spatial intratumoral heterogeneity that is critical for MB progression, relapse and treatment resistance (Tech et al., 2017; Hovestadt et al., 2019; Ocasio et al., 2019; Zhang et al., 2019; Luo et al., 2021). Given the marked heterogeneity among MBs, personalizing therapy to a patient’s tumor subtype and individual genetic, epigenetic and transcriptomic characteristics constitute the next frontier in oncology. Tumor heterogeneity is, at least in part, justified by the presence of cancer stem cells (CSCs) that represent a small subgroup of cells in the tumor bulk with stem-like features, recognized to be responsible for tumor onset, maintenance, and relapse after therapy. CSCs have been identified in many types of brain tumors, including glioblastoma (GBM) and MB; they are able to self-renew under clonal conditions, and differentiate into neuron- and glia-like cells as well as into atypical cells with mixed phenotypes (Singh et al., 2004). Many findings suggest that enriched, treatment-resistant subpopulations of CSCs are involved in driving MB tumor recurrence and metastasis subsequent to standard treatment (Sun et al., 2017; Bakhshinyan et al., 2021).

Metabolic adaptation is believed to be one of the hallmarks of tumor cells and several factors (both cell-intrinsic, such as oncogenes and tumor suppressor genes, and cell-extrinsic such as nutrient availability or hypoxia) contribute towards driving metabolic reprogramming. Normal cells usually convert glucose into pyruvate that has two possible fates mainly depending on the availability of oxygen. Under aerobic conditions, the glycolytic product pyruvate is mostly oxidized in the tricarboxylic acid cycle (TCA cycle; also known as the Krebs cycle) to produce H_2_O, CO_2_ and secondary metabolites; these act as electron donors in the oxygen-dependent mitochondrial oxidative phosphorylation (OXPHOS), a process which is coupled with high ATP production rate. Under hypoxic conditions, pyruvate undergoes anaerobic fermentation, giving lactate and concomitantly producing two molecules of ATP per molecule of glucose. The aim of the latter two processes (OXPHOS and lactic acid fermentation) is the recycling of co-enzymes for further glycolysis and other cellular enzymatic activities. On the contrary, cancer cells preferentially rely on glycolysis and lactic acid fermentation rather than OXPHOS to produce ATP, even in the presence of O_2_ (Warburg, 1956; Racker and Spector, 1981). This metabolic reprogramming is known as the Warburg effect or “aerobic glycolysis”.

Besides glycolysis, fatty acid (FA) biosynthesis and glutamine metabolism are two other important metabolic pathways altered in both cancer cells and CSCs. Indeed, cancer cells use the process of *de novo* lipogenesis to convert carbohydrates into FAs and lipids and alterations in this process are associated with increased proliferation and spreading (Koundouros and Poulogiannis, 2020). Lastly, according to recent studies, CSCs rely on the metabolism of the amino acid glutamine, which provides the carbon and the amino-nitrogen needed for the biosynthesis of amino acids, nucleotides, and lipids.

It is important to highlight that cellular heterogeneity inside the tumors may result in different metabolic requirements, and consequently, in different responses to metabolic therapies. In particular, the maintenance of stem cell functions is, at least in part, regulated by metabolic alterations that initially consist of the activation of glycolysis and the inhibition of OXPHOS. Despite the majority of data suggesting that CSCs are primarily glycolytic and that they are usually characterized by the upregulation of glycolytic enzymes, glucose uptake and lactate production, mounting evidence also shows their capability to exploit the mitochondrial oxidative metabolism.

**FIGURE 1:**
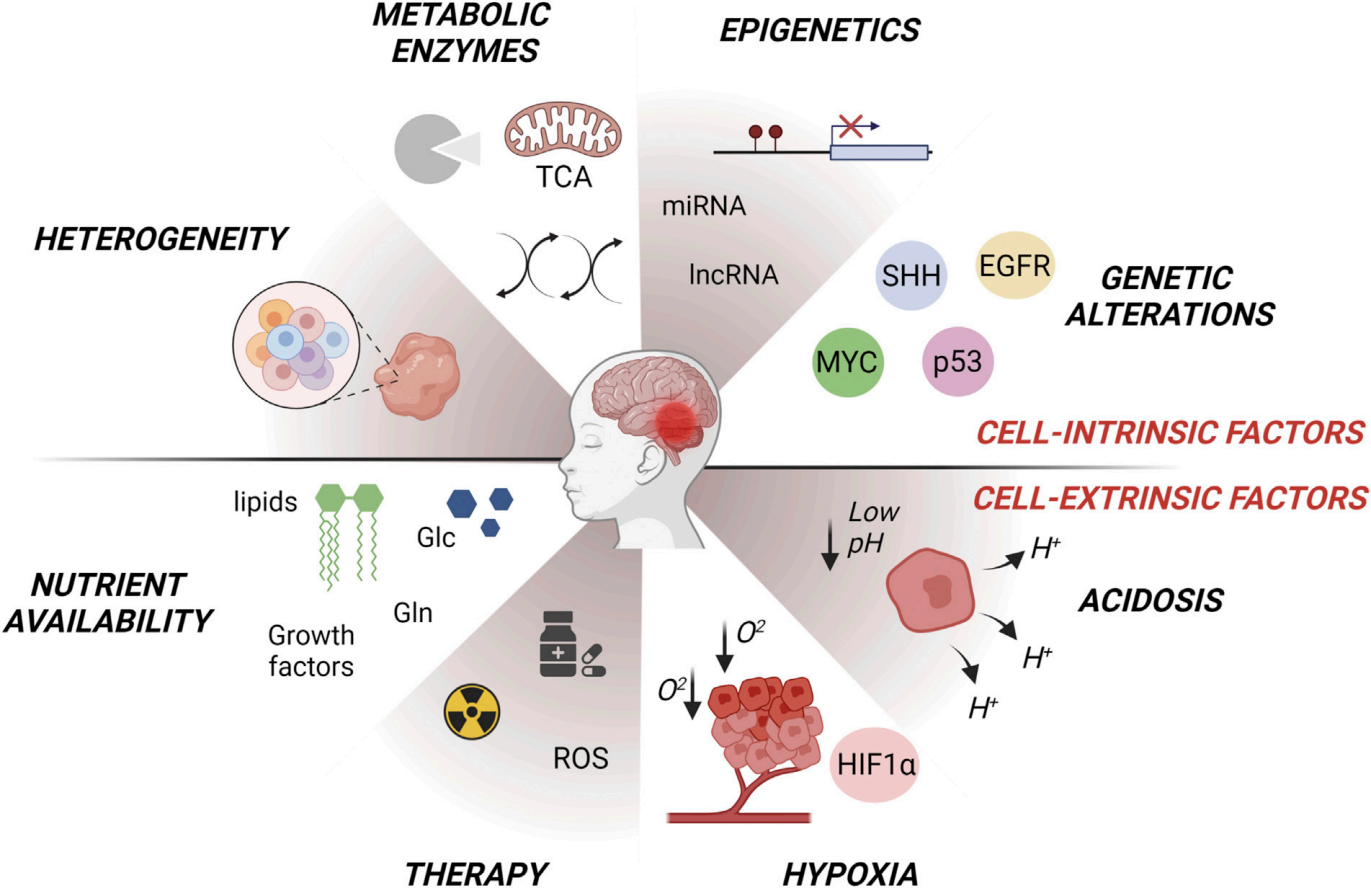
Intrinsic and extrinsic factors affecting metabolic changes in MB subgroups (WNT, SHH, Group3, Group4). Tumor intrinsic factors include heterogeneous oncogenic signaling and/or genetic alterations in metabolic enzymes that modulate gene expression through a variety of mechanisms such as signal transduction and epigenetic reprogramming. Tumor extrinsic factors consist of microenvironmental availability of nutrients such as glutamine or amino acids, oxygen and changes in extracellular pH. Alterations in metabolism influence glycolysis, lipogenesis, OXPHOS, oxygen reactive species (ROS) production, glutamine-dependent signaling and tumor heterogeneity, giving rise to metabolic adaptations. Figure is created in “BioRender.com”.

This review summarizes the latest knowledge regarding MB-specific metabolic alterations at the cellular and the molecular level (Figure 1). Unraveling the metabolic networks of both tumor cells and CSCs in MB could be helpful for designing new targeted therapies that interfere with energy cell metabolism, with the potential to minimize relapse and resistance after treatments.

## Metabolic reprogramming in medulloblastoma

In the last decades, several works have focused on the role of metabolic rewiring in brain tumors such as GBM (Tardito et al., 2015; Bi et al., 2020), whereas our comprehension about metabolic features underlying MB pathogenesis and progression still remains limited. It has been shown that metabolic changes during cancer development are similar to the ones that occur during neural development: neurogenesis, indeed, requires an increase in spatiotemporal-regulated proliferation as it occurs in cancer. Many studies have shown that SHH-driven MB metabolic features largely mirror cerebellar granule precursors (CGPCs) phenotype. Aberrant SHH pathway activation induces an upregulation of aerobic glycolysis and lipogenesis, while decreasing fatty acid oxidation levels, as it occurs in CGPCs (Tech et al., 2015; Tech and Gershon, 2015). Nonetheless, since the SHH MB subgroup accounts for approximately one-third of MB diagnoses, it is of paramount importance to expand our knowledge about the other MB subgroups that likely arise from different tumor-initiating populations (Hovestadt et al., 2020). A large-scale bioinformatic analysis using data of 530 patients from the Medulloblastoma Advanced Genomic International Consortium (MAGIC) project has identified cancer metabolism-related pathways as new prognostic factors in all MB subgroups (Park et al., 2019). This work has allowed the identification of subgroup-specific metabolic signatures that could be targetable to improve the clinical outcome of MB patients. Park and others demonstrated that several prognostic genes involved in the glycolytic pathway (such as *ENO1*, encoding enolase 1) and lipogenesis (such as *FASN* and *SCD* encoding fatty acid synthase and stearoyl-CoA desaturase, respectively) are upregulated in three MB subgroups (SHH, Group 3, and Group 4). Specifically, glycolysis and glutaminolysis have been identified as prognostic or highly expressed in both SHH and Group 3 subgroups, suggesting that ‘Warburg effect’ may drive MB energy metabolism. One carbon (folate) cycle or serine synthesis (that are commonly used for nucleotide synthesis, methylation and reductive metabolism supporting the high proliferative rate of cancer cells), are mainly prognostic in both SHH and Group 4 subgroups. In addition, MYC-driven Group 3 MB are marked by pentose phosphate pathway (PPP) upregulation, which is frequently boosted by cancer cells to provide nucleic acids and nicotinamide-adenine dinucleotide phosphate (NADPH) for cell growth and survival under stressful conditions (Park et al., 2019). Yet, more research is needed to determine whether and how the identified metabolic signatures cross-talk with the genetic heterogeneity across MB subgroups.

***Glucose metabolism and aerobic glycolysis***. Glucose metabolism provides the fuel for physiological brain function. In neurons, glucose enters through the sodium-independent facilitative transporters GLUT1 and GLUT3 (encoded by *SLC2A1* and *SLC2A3* genes respectively), which are differentially distributed across the brain. GLUT1 is upregulated in embryonal tumors including MB (Loda et al., 2000), with a prominent expression in MYC-driven Group 3 MB (Park et al., 2019; Pham et al., 2022). GLUT1 overexpression determines a higher glucose influx into the cell that is avidly used for cytoplasmic glycolysis, supporting cell survival in low oxygen environments (Al Tameemi et al., 2019). As soon as glucose enter the cells, hexokinase converts glucose into glucose-6-phosphate (G6P) which is consumed by multiple pathways such as glycolysis, OXPHOS, PPP, hexosamine biosynthetic pathway (HBP) or amino acid biosynthesis. By using (18F)-fluorodeoxyglucose positron emission tomography (PET), a glycolytic metabolic phenotype has been reported in MB patients (Gururangan et al., 2004), and key genes involved in glycolysis are found to be significantly overexpressed in MYC-driven MB mouse models compared to SHH MB or non-neoplastic cerebellar tissue (Tao et al., 2019). Furthermore, both SHH MB and proliferating cells of the postnatal brain (i.e., hippocampus, subventricular zone, cerebellar external granule layer) preferentially express the glycolytic enzymes Hexokinase 2 (*HK2*) and the M2 isoform of pyruvate kinase (*PKM2*) (Gershon et al., 2013a, 2013b; Di Magno et al., 2014; Tech et al., 2017). HK2 and PKM2 are both key glycolysis rate-limiting enzymes, also in the presence of normal oxygen rate. Intriguingly, there are four mammalian hexokinases that vary in their tissue distribution and affinity for glucose; among them, HK2 drives aerobic glycolysis in MB (Tech et al., 2017). Moreover, pyruvate kinases catalyze the latest step of glycolysis: the M1 isoform (PKM1) is typically present in differentiated neurons, whereas high PKM2 expression characterizes all MB subgroups (Tech et al., 2017).

Under aerobic conditions, pyruvate could be converted into lactate by the enzyme lactate dehydrogenase (LDH), and this reaction is coupled with NAD+ regeneration. Lactate can be exported outside the cell and contributes to both intra- and extracellular acidification. High lactate levels have been primarily associated with Group 3/4 compared to SHH MB (Blüml et al., 2016), and MB Group 3 orthotopic mouse models also exhibit high lactate levels (Pham et al., 2022). Moreover, lactate dehydrogenase A (LDHA) levels are found overexpressed in the most aggressive MB subgroups (Valvona and Fillmore, 2018). Of interest, monocarboxylate transporter 1 (MCT1, encoded by the *SLC16A1* gene), which mediates the import and export of lactate, pyruvate and ketone bodies across the plasma membrane, is upregulated in MB tumors and cell lines (Li et al., 2009). Supporting these findings, miRNA 124 (miR124), an important regulator of the temporal progression of neurogenesis in mice, targets MCT1 and it is downregulated in MB tumors (Li et al., 2009; Silber et al., 2013). On the other hand, c-MYC is reported to be a positive modulator of MCT1 (Coller et al., 2000), suggesting that *c-MYC* amplification or overexpression could contribute to enhance MCT1 expression levels in MB. These findings raise the possibility to target the glycolytic pathway as a novel therapeutic strategy against MB.

Interestingly, a comparative analysis of metabolic profiles of MB cells in three different environments -*in vitro*, in flank and in orthotopic xenografts- suggests that orthotopic MYC-amplified MB tumors have increased levels of glucosamine-6-phosphate compared to normal brain, proposing dependence on HBP (Pham et al., 2022). Similarly, RNA-sequencing data derived by the “Children’s Brain Tumor Network/Kids First Pediatric Brain Tumor Atlas'' indicate an increase in HBP enzymes expression in MB tumors compared to other pediatric brain tumors (i.e., glioma, ependymoma) (Pham et al., 2022).

***Glutamine addiction.*** Glutamine is a nonessential amino acid (NEAA) implicated in different processes of cell proliferation, acting as carbon and nitrogen source for the biosynthesis of many other molecules such as nucleotides, other nonessential amino acids (alanine, aspartate, asparagine, glycine, etc), glutathione (GSH) and metabolic TCA intermediates (Zhang et al., 2017; Yoo et al., 2020). Glutamine as a precursor for the neurotransmitter glutamate plays a crucial role during the development of the nervous system. In normal conditions, glutamine is metabolized in the cytosol for the biosynthesis of nucleotides and amino acids and can also be transported into the mitochondria for glutaminolysis. The latter is a two-step process: the first reaction is the hydrolysis of glutamine in glutamate that is catabolized by glutaminases (GLS) 1/2, also known as glutamine synthetases; then glutamate is deaminated by the glutamate dehydrogenases (GDH) to produce α-ketoglutarate (α-KG) that supplies the TCA cycle. Additionally, glutamate undergoes expulsion into cytoplasm for generating GSH and other NAAs (Yoo et al., 2020). However, as also noted, glutaminase II pathway converts glutamine directly into α-KG by glutamine transaminase (GTK, and by ω-amidase). The latter has recently been reported as the main pathway implicated in the consumption of glutamine in the brain as well in primary Group 3 cells and MB mouse models (Pham et al., 2022).

Additional implications relevant to glutamine metabolism in MB have emerged. A role for p73 as a regulator of glutamine metabolism has been reported in MB (Velletri et al., 2013; Niklison-Chirou et al., 2017). P73 is a member of the p53-family, which is frequently overexpressed in different tumors, including MB (Zitterbart et al., 2007). In the most aggressive subgroups of MB, Niklison-Chirou and others reported high levels of TAp73, the p73 form that maintains the transactivation domain. TAp73 was previously recognized as a positive modulator of energetic metabolism by enhancing PPP (Jiang et al., 2013), inducing cytochrome c oxidase (Rufini et al., 2012), and activating the serine biosynthesis (Amelio et al., 2014). Additionally, high glutamine uptake and glutaminase activity are found to be upregulated in cisplatin-resistant MB cell line DAOY (Ge et al., 2022). Interestingly, the NEAT1 (non-coding RNA nuclear paraspeckle assembly transcript 1)/miR-23a-3p/GLS axis has emerged as a master regulator of glutamine metabolism in MB by regulating GLS activity and mitochondrial glutaminolysis. In both MB patients and in cisplatin-resistant DAOY cells, miR-23a-3p was downregulated, while the expression levels of NEAT1 were upregulated (Ge et al., 2022). Since cancer tissue of origin influences cellular metabolic reprogramming, the dependence of cancer cells on glutamine remains difficult to elucidate. For instance, GBM showed a high demand of glutamine, which is reported to be synthesized by GBM cells themselves and in part released by astrocytes in the cerebrospinal fluid (CSF). GBM cells convert glutamate to glutamine to sustain amino acid and nucleotide *de novo* biosynthesis, thus providing metabolites for TCA and OXPHOS (Tardito et al., 2015). A recent comprehensive metabolic study investigated the metabolic profiles of MYC-amplified MB Group 3 cell lines and different mouse models using labeled glucose. A high uptake of glucose in normal brain and orthotopic xenografts compared to *in vitro* cell cultures was found, suggesting that MYC-amplified MB use glucose as carbon source for the glutamate synthesis (deriving by TCA intermediates), depending on the tumor environmental conditions (Pham et al., 2022). These findings corroborate the hypothesis that high-risk MB tumors do not acquire glutamine by the TME but are able to synthesize it *de novo* using glucose.

As reported above, addiction to glutamine is exploited by cancer cells to produce GSH as antioxidant defense (Bansal and Celeste Simon, 2018) and several works now focus on the efficacy of targeting GLS to improve cancer therapy. High levels of GLS were associated with low reactive oxygen species (ROS) levels due to increased antioxidant response in cancer. Interestingly, a novel glutamine antagonist JHU395 was shown to inhibit GLS and to selectively kill MYC-driven MB, supporting the idea that ROS increase underlies GLS inhibition-induced cytotoxicity (Pham et al., 2021). C-MYC is a critical regulator of glutamine uptake by regulating the glutamine transporters SLC1A5 (also known as ASCT2). A work from Genovesi et al., identified SLC1A5 in a set of potentially druggable-targets in non-WNT restricted MB through a network-based system-pharmacogenomics approach (Ghasemi et al., 2022). Since c-MYC deregulation is frequent in non-WNT highly aggressive and metastatic MB subtypes, it is possible that SLC1A5 inhibition could induce high-risk MB regression by interfering with both glutamine catabolism and ROS scavenging, boosting ROS levels beyond the tolerable threshold. It is reasonable to hypothesize that targeting glutamine metabolism may impact on MB survival by both limiting the availability of critical metabolites and heightening oxidative stress in MYC-deregulated aggressive MB.

Using *in-vivo* Magnetic Resonance Spectroscopy (MRS), glutamate concentration has emerged as a survival prognostic marker of MB and elevated glutamate levels are reported in high-risk MB cases (Wilson et al., 2014). Glutamate decarboxylases (GAD1/2) are the enzyme responsible for the conversion of glutamate into the inhibitory neurotransmitter γ-aminobutyric acid (GABA) (Grone and Maruska, 2016). An increase in both GABA uptake and GABA metabolism is found in brain metastases derived from breast cancer, and an increase of NADH levels in the microenvironment is linked to this event (Neman et al., 2014).

Recent findings revealed GAD2 overexpression in primary MB Group 3 D425 cell line when cultured in CSF-mimicking condition, indicating *de novo* GABA biosynthesis. On the contrary, GABA catabolism is mediated by 4-aminobutyrate aminotransferase (ABAT, or GABA transaminase) as a shunt of energy and glutamine under nutrient deprivation and environmental stresses (Araújo et al., 2010; Feehily and Karatzas, 2013). Of interest, while differentiated neurons show both high OXPHOS rate and ABAT expression, neural stem cells and MB tumors exhibit low ABAT levels; notably, ABAT levels are lower in the metastatic G3/G4 subgroups compared to the less aggressive WNT/SHH subtypes. Intriguingly, during dissemination, MB cells exploit GABA metabolism to survive in the nutrient-poor environment of CSF, facilitating metastasis formation; this suggests a crucial role for ABAT expression fluctuation to promote leptomeningeal dissemination (Martirosian et al., 2021).

***Lipogenesis*** *.* Lipid metabolism has been well-characterized in SHH MB, as SHH signaling suppresses fatty acid oxidation and exacerbates lipogenesis and aerobic glycolysis (Tech et al., 2015; Tech and Gershon, 2015). Mechanistically, the crucial lipid synthesis enzymes FASN and Acetyl-Coenzyme A (Acetyl-CoA) Carboxylase (Acc1) are upregulated by SHH MB, whereas lipid catabolism enzymes Acyl-Coenzyme A Oxidase 1 (Acox1) and Medium Chain Acyl-CoA Dehydrogenase (MCAD) are downregulated in the same MB subgroup (Bhatia et al., 2011). Accordingly, high lipid levels were found in SHH MB tissues compared to Group 3/4 (Blüml et al., 2016). The SHH signaling also drives lipid metabolic changes through the retinoblastoma protein (Rb)/E2F1 tumor suppressor complex inactivation in both CGNPs and SHH-driven MB (Bhatia et al., 2011). Under this condition, E2F1 up-regulates the nuclear nutrient sensor Peroxisome Proliferator-Activated Receptor-γ (PPAR-γ), which positively regulates the expression of glycolytic enzymes HK2 and PKM2 (Bhatia et al., 2011, 2012).

A double regulation mechanism connecting SHH signaling and lipogenesis has recently emerged. Although SHH signaling drives lipogenesis, endogenous lipids can lead to SHH hyperactivation through the binding and the modulation of the SHH-activating key component Smoothened (SMO) protein (Deshpande et al., 2019; Daggubati et al., 2021). Endogenous sterol and oxysterol lipids (i.e., HSD11β2 and DHCR7) are indeed reported to activate SMO after ribosome biogenesis impairment, which consequently induces ER stress and unfolded protein response activation (Raleigh et al., 2018; Daggubati et al., 2021). Cholesterol biosynthesis is also found enhanced in SHH MB tumors as it promotes SMO activation (Gordon et al., 2018). Of interest, treatment with the current Food and Drug Administration (FDA)-approved SMO antagonist (i.e. Vismodegib and sonidegib) gives rise to drug resistance development in SHH MB patients due to several mechanisms, including SMO re-activating mutations. Statin treatment by simvastatin reduces SHH MB cells proliferation and SHH-derived MB young mice tumor growth (Fan et al., 2021), as single agents or combined with SMO antagonists (Gordon et al., 2018; Fan et al., 2021). These findings suggest that targeting lipid metabolism in association with current chemotherapy could represent a promising strategy to overcome SHH MB recurrence. However, further studies are required to delve into the role of fatty acid metabolism in all MB subgroups.

### Metabolic adaptation induced by cell-intrinsic factors

**Genetic-driven metabolic adaptation.** As previously described, genetic diversity is one of the major drivers of metabolic adaptation in MB. For this reason, mutational signatures specific to each subgroup may underlie metabolic heterogeneity across MB subgroups. Mutations affecting oncogenes or tumor suppressor genes could act as cell-intrinsic factors regulating metabolic functions in cancer cells to support the synthesis of specific metabolites in a context-dependent manner.

**FIGURE 2:**
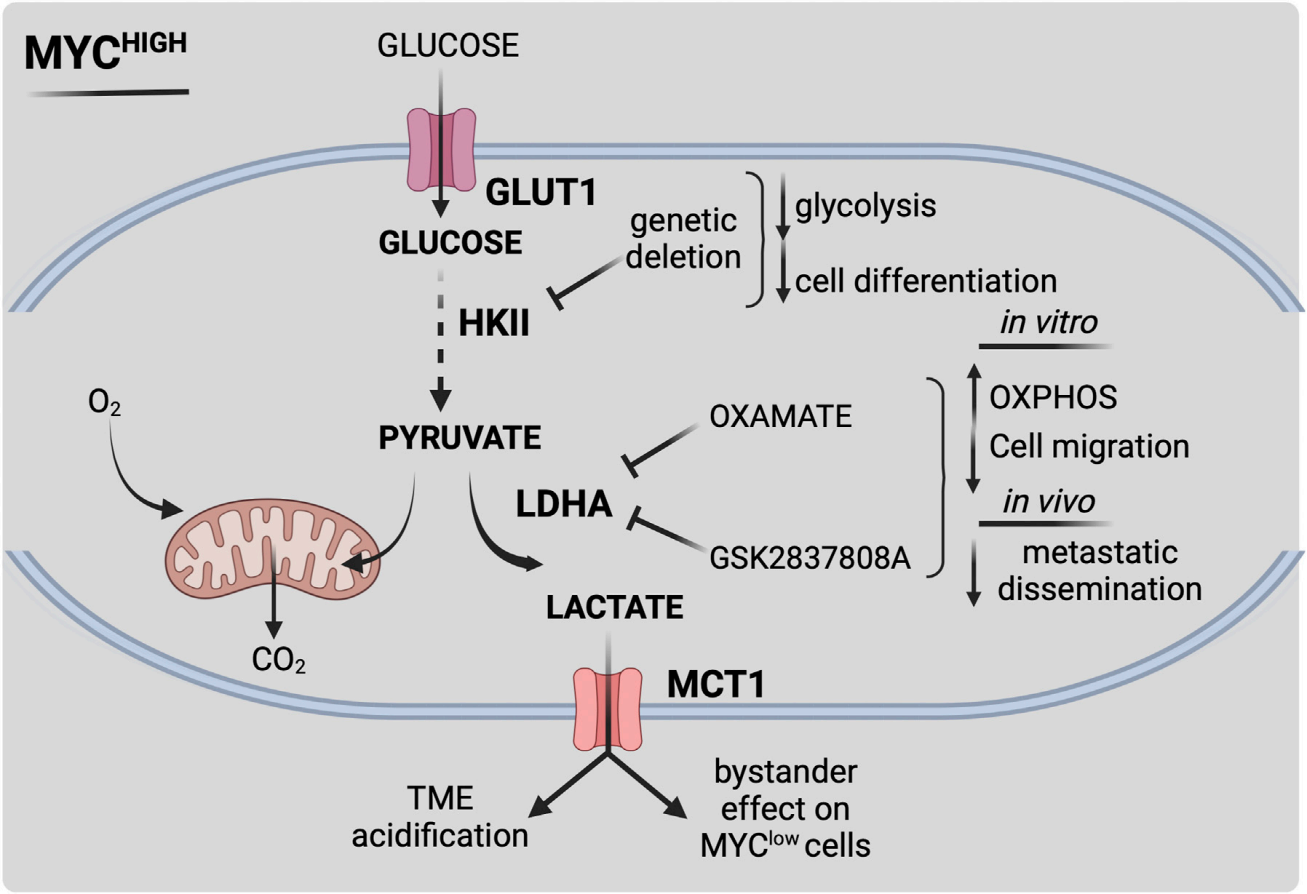
Schematic representation of MYC-dependent metabolic reprogramming in MB. MYC overexpression or amplification (MYC^high^) drives metabolic reprogramming, leading to increased survival and therapy resistance. MYC-dependent alterations lead to enhanced aerobic glycolysis (i.e., Warburg effect) by a multiple-layered regulation. Activation of MYC stimulates glucose uptake by increasing transcription levels of glucose transporter GLUT1. MYC-dependent upregulation of hexokinase 2 (HKII) and pyruvate dehydrogenase kinase 1 (PDK1) fuels glucose flux within the cell. MYC highly contributes to extracellular acidification by acting on lactate synthesis and secretion through lactate dehydrogenase A (LDHA) and monocarboxylate transporter 1 (MCT1) expression, thus influencing MB microenvironment. Pharmacological targeting of MYC-dependent key metabolic reactions could be effective towards high-risk MYC-driven MB. Figure is created in “BioRender.com”.

***MYC***. The oncogene *MYC* is the most studied gene driving MB progression and metastasization and it is frequently amplified or overexpressed in MB, especially in high-risk Group 3 (McManamy et al., 2007; Pfister et al., 2009; Cho et al., 2011; Northcott et al., 2012a, 2012b). However, since MYC was frequently found upregulated also in patients with good prognosis, the relationship between MYC expression and MB clinical outcome remains controversial. That is why it is becoming increasingly evident that MYC *per se* cannot be considered a good prognostic factor (Roussel and Robinson, 2013). These discrepancies might be explained by the presence of several determinants, both intrinsic (e.g. mutational co-dependencies, metabolic plasticity) or environmental (e.g. tumor microenvironment), that can influence MYC-related prognosis in MB patients. A large body of evidence supports the idea that MYC can profoundly shape the metabolic state of cancer cells by regulating a plethora of processes such as glutamine synthesis, glycolysis and lipogenesis as well as mitochondrial biogenesis and metabolism. Although inhibiting MYC proteins could ideally be a powerful approach to eradicate MB, this strategy results in low effectiveness in clinical settings, due to the highly flexible structure of MYC proteins and to the lack of drug binding pockets. A more promising therapeutic avenue encompasses the possibility of targeting pathways that are activated downstream of this oncoprotein; in this regard, MYC-dependent metabolic adaptation is a very attractive phenomenon with several potential novel targets for MB (Figure 2). As it occurs in other cancers, MYC stimulates the Warburg effect in MB by directly trans-activating the expression of key glycolytic genes (Tao et al., 2019). Several crucial metabolism-related genes are *bona fide* MYC targets both in healthy and in transformed cells. It has been demonstrated that MYC overexpression in astrocyte precursors is able to induce neoplastic formation, phenocopying Group 3 MB in mice. RNA-sequencing analyses of MYC-driven Group 3 mouse models revealed an upregulation of pro-glycolytic MYC-targets such as LDHA, PKM2, HK2 and Pyruvate dehydrogenase kinase 1 (PDK1) compared to normal cerebellum or SHH MB (Tao et al., 2019). This evidence supports the idea that MYC is a prominent positive regulator of the Warburg effect in MB. Interrogation of Gilbertson MB public dataset (Tumor MB-Gilbertson- 76–MAS5.0–u133p2 dataset) showed that LDHA mRNA is upregulated in WNT and Group 3 MB (Valvona and Fillmore, 2018) compared to non-neoplastic cerebellum and spinal cord tissue. LDHA was also found over-expressed *in vitro* both at the transcriptional and at the protein level in DAOY (SHH), UW402 (Group 3) and Res256 (subgroup non-attributed) MB cell lines (Valvona and Fillmore, 2018). LDHA inhibition through treatment with oxamate, a structural analogue of pyruvate, reduces MB glycolysis in favor of OXPHOS and blocks cell migration *in vitro* (Valvona and Fillmore, 2018). A very interesting question is whether and how and the intra-tumor heterogeneous expression of MYC contributes to MB progression and relapse. To this aim, a recent work has identified a functional cross-talk between *MYC* amplified and non-amplified cells within MB both *in vitro* and *in vivo* (Qin et al., 2022). By single-cell transcriptomics and mass-spectrometry-based metabolite quantification, the authors identified a prominent role for inter-clonal communication through secreted factors in the determination of MB aggressiveness (Figure 2). On one hand MYC-driven MB clones increased metastasizing capacity and leptomeningeal dissemination through LDHA release, while non-MYC-driven cells secreted Dickkopf WNT signaling pathway inhibitor three to drive neo-angiogenesis (Qin et al., 2022). The authors also found that LDHA secretion from MYC-driven cells in the tumor stroma is able to induce the conversion of lactate in non-MYC-driven cells, increasing their migratory propensity as a by-stander effect; this strengthens the notion that cell-to-cell communication may reinforce cancer aggressiveness. Moreover, pharmacological targeting of LDHA with GSK 2837808A reduces MB cell invasion *in vitro* prompting a highly relevant therapeutic opportunity associated with LDHA targeting (Qin et al., 2022).

A previously reported role for a c-MYC paralog, N-MYC, as an oncometabolic regulator has been established in SHH MB. N-MYC acts as a mediator of SHH/insulin/IGF/PI3K signaling pathways, leading to the final upregulation of HK2. Interference of N-MYC activity with the 10,058-F4 inhibitor leads to the impairment of SHH-induced HK2 upregulation in CGPCs (Gershon et al., 2013a; 2013b). Similarly, upon oncogenic SHH expression, HK2 deletion blocks glycolysis and induces cell differentiation while disrupting tumor growth (Gershon et al., 2013a; 2013b). These findings firstly corroborate the idea of cancer cells exploiting the energy metabolism of their developmental progenitors to maintain an undifferentiated state and a malignant potential. Moreover, they support the central role of MYC as master regulator of MB malignant phenotype and suggest its downstream effectors as potential novel targets and biomarkers for metabolic anticancer therapy.

***P53*.** P53 is the most studied tumor suppressor, whose genetic inactivation has been extensively linked to the pathogenesis of the majority of human cancers. Beyond its well-known role as genome caretaker, p53 was also found to be a critical regulator of metabolic adaptation in several cancer types, including embryonal brain tumors (Marie and Shinjo, 2011; Xiong et al., 2020). At the same time, p53 is shown to be promptly activated by metabolic stresses. In human MBs, TP53 mutations are frequent in the WNT (16%) and SHH groups (21%), acting as promoter of cellular transformation, and are rare in Group 3 or 4 tumors (Zhukova et al., 2013). Although p53 genetic mutations are rare in MB with the poorest prognosis, a body of evidence suggests that p53 pathway is perturbed in high-risk MB. Even if the role of p53 is well-characterized in the SHH MB subgroup, its involvement in metabolic MB reprogramming is completely unexplored. Genomic data analysis from MB have shown that overexpression of the p53-antagonist gene WIP1 (wild-type p53-induced phosphatase one or protein phosphatase, magnesium-dependent 1, delta, PPM1D) is crucial to counteract p53-mediated apoptosis, thus mediating therapy resistance (Castellino et al., 2008; Buss et al., 2012, 2014; Akamandisa et al., 2016). Interestingly, WIP1 function can be regulated by phosphoglycerate mutase 1 (PGAM1), a glycolytic enzyme that is overexpressed in cancer tissues and that is able to block WIP1 cytoprotective function upon chemo- and radiotherapy in glioma (Ohba et al., 2020). These data highlight a key role for p53-related functions in the intricate crosstalk between cancer metabolic rewiring and therapy responsiveness, which could also be relevant for MB. An intriguing work studied the biological alterations of MB at relapse with respect to the primary tumors in patients and through *in vivo* studies (Hill et al., 2015). The authors strikingly found that combined MYC family amplifications and p53 pathway defects commonly emerged at relapse, and all patients in this group died of rapidly progressive post-relapse disease. This work contributed to elucidate an unprecedented role for novel co-occurring MYC/p53 mutations as determinants of dismal prognosis in MB (Hill et al., 2015). Since MYC amplification and p53 inactivation drive cell metabolism in the same direction, it is possible that MB stem cells acquire and exploit this second mutation to enhance their metabolic plasticity under the selective pressure of their microenvironment and drive metastases formation.

Many lines of evidence suggest that TIGAR (TP53-induced glycolysis and apoptosis regulator), a downstream target of p53, could fill the knowledge gap concerning P53 and metabolic plasticity in MB. TIGAR mainly functions as a glycolytic inhibitor by mediating the hydrolysis of fructose-1,6-diphosphate and fructose-2,6-diphosphate to inhibit glycolysis. While counteracting glycolysis, TIGAR facilitates PPP flux to produce NADPH and ribose, thereby promoting DNA repair, and reducing intracellular ROS (Lee et al., 2014). In recent years, TIGAR enzyme was shown to actively control cancer cell differentiation, mitochondrial function and autophagy (Geng et al., 2018) and it was found to be overexpressed in MB specimens (Li et al., 2012). Given its role in other tumors, TIGAR could antagonize p53 and promote PPP in a subpopulation of MB cells to sustain proliferation and antioxidant defenses during MB dissemination through the CSF. Moreover, it might be interesting to estimate TIGAR activity in MB and measure its metabolic-dependent contribution to therapy resistance.

***EGFR.*** Very well-recognized oncogenic drivers are the members of the family receptor tyrosine kinase (RTKs) which actively support metabolic adaptation during cancer progression. Erb-B2 Receptor Tyrosine Kinase 2 **(**ERBB-2) is a member of the human epidermal growth factor receptor (EGFR) family and it is of particular relevance in MB; it is upregulated in approximately 40% of MB and its expression correlates with poor prognosis in patients (Bodey et al., 2005; Liu et al., 2014).

In several cancer settings, constitutive EGFR activation has been linked to increased glucose uptake, lactate secretion and glutamine utilization (Makinoshima et al., 2014; Sigismund et al., 2018). Additionally, it was shown that EGFR activation correlates with poor prognosis in MB patients and is a key determinant of MB migratory behavior (Rico-Varela et al., 2015). The contribution of EGFR activation to metabolic reprogramming in MB was however never explored directly. It was recently unveiled that EGFR oncogenic mutations can cause a switch from glycolysis towards the serine biosynthetic pathway (Jin et al., 2019), which was found enhanced in Group 4 MB (Park et al., 2019). In line with this observation, it could be relevant to explore whether this metabolic feature represents a specific metabolic vulnerability that could be exploited to hit EGFR-driven MB. In this respect, inhibition of 3-phosphoglycerate dehydrogenase (PHGDH), the rate limiting enzyme for *de novo* serine biosynthesis, turned out to be a promising therapeutic strategy to overcome resistance in metabolically-driven cancers (Rathore et al., 2020, 2021; McNamee et al., 2021; Zhao et al., 2021). Moreover, PHGDH inhibition was shown to be particularly effective in relapsing and therapy resistant tumors, as it was demonstrated for reduced brain metastasization (Ngo et al., 2020). It is plausible that upregulation of PHGDH-dependent serine/glycine biosynthesis supports EGFR-driven MB metabolic adaptation during leptomeningeal colonization and therefore PHGDH could be a potential target for metastatic MBs. Moreover, stratification of MB patients based on the expression of EGFR could help develop tailored metabolic therapies.

**Epigenetic-driven metabolic adaptation.** Amongst cell-intrinsic factors epigenetic alterations can shape the transcriptional program in order to allow cancer adaptation to the increased metabolic requirements. In MB, the interplay among cell metabolism and epigenetic regulation of gene expression is very poorly characterized. Mutations that typically arise in MB patients involve chromatin remodeling factors genes such as AT-Rich Interaction Domain 1A/2 (ARID1A, ARID2) and SWI/SNF Related, Matrix Associated, Actin Dependent Regulator Of Chromatin, Subfamily A, Member 4 (SMARCA4) (Morrissy et al., 2017). SMARCA4 mutations in WNT, Group 3 and Group 4 MB subgroups are reported and are found to counteract MYC activity in Group 3 MB (Morrissy et al., 2017; Ballabio et al., 2021). Moreover, in other cancers, SMARCA4 and ARID1A are found to repress the transcription of the sodium independent anionic amino acid transporter SLC7A11 (Koppula et al., 2018; Ogiwara et al., 2019; Song et al., 2020; Luo et al., 2021; Kito et al., 2022). Other than exporting glutamate outside the cell membrane, SLC7A11 imports cysteine, which is essential for GSH biosynthesis. Indeed, SMARCA4-dependent negative regulation of this transporter leads to reduction in intracellular cysteine required for antioxidant defense (Koppula et al., 2018; Song et al., 2020; Cermakova et al., 2021). Thus, loss-of-function mutations in the *SMARCA4* gene could drive MB survival under oxidative stress conditions. Latest findings have unraveled new mechanisms linking epigenetics and metabolism in Group 4 MB. Badodi and others identified a cellular subset of Group 4 MB characterized by high expression of BMI1, a member of the Polycomb-Group Proteins, and low expression of the chromatin remodeler CHD7 (herein referred to as BMI1High; CHD7Low) both *in vitro* and in MB patients, respectively. This cellular population was shown to support MB growth (Badodi et al., 2017) through an overall decrease in mitochondrial function combined with upregulation of glycolysis (Badodi et al., 2021). Such mutated genetic background characterized by chromatin alterations is shown to favor the establishment of the Warburg effect as an adaptive strategy. Inositol hexakisphosphate (IP6) treatment is able to dramatically reduce glycolysis in the BMI1High; CHD7Low subpopulation without activating OXPHOS; it also synergizes with cisplatin to induce MB Group 4 clearance both *in vitro* and *in vivo* (Badodi et al., 2021). Given the role of both BMI1 and CHD7 in low oxygen cellular conditions, it is conceivable that this signature identifies a proportion of Group 4 MB with a specific advantage in hypoxic conditions due to the described metabolic adaptations. Another understudied layer of regulation of MB metabolic plasticity is represented by the plethora of non-coding RNAs affecting the expression of key metabolic genes, of which some are found deregulated in MB. An example of this epigenetic-dependent metabolic regulation in MB is provided by the discovery of a crosstalk between lncRNA NEAT1 and miRNA-23a-3p (miR-23a-3p) in the regulation of glutamine metabolism, which supports cisplatin resistance in MB cell lines (Ge et al., 2022). Specifically, NEAT1 is reported to promote therapy-resistance through the repression of miR-23a-3p expression, which is extremely low in MB samples. The authors have also demonstrated that miR-23a-3p exerts a tumor suppressive function in MB by targeting the gene encoding for GLS, thus attenuating glutamine metabolism. Therapeutic strategies enhancing miR-23a-3p expression should be validated *in vivo* to explore the possibility to target glutamine metabolism in cisplatin-refractory MB. Another miRNA that is implicated in MB metabolic reprogramming is microRNA 124 (miR124), whose function during neurogenesis was previously studied (Cheng et al., 2009). miR124 is significantly downregulated in MB (Li et al., 2009; Silber et al., 2013) and is found to control the expression of the main lactate transporter MCT1 (SLC16A1). Restoration of miR124 by ectopic expression induces MB cell death through the inhibition of lactate secretion by MCT1, probably due to cytotoxic intracellular acidosis.

**Interplay between metabolic reprogramming and ROS production.** In physiological conditions, ROS play a key role as a messenger in cell signal transduction and cell cycling while an increase in their production generates the so-called ‘oxidative stress’, that is widely considered as a fuel for tumorigenesis (Perillo et al., 2020; Cheung and Vousden, 2022). Cancer cells maintain low levels of ROS in two essential ways: by enhancing their antioxidant capacity (i.e. increase of NADPH and GSH) and/or by reducing the reliance on mitochondrial OXPHOS (Liemburg-Apers et al., 2015; Lee et al., 2018). It is now clear that altered bioenergetics affects ROS production, which is in turn able to regulate cancer metabolism through the control of key metabolic enzymes (Arfin et al., 2021). Little is known about the role of oxidative stress in the development and the evolution of MB. Given the ability of CSCs to maintain extremely low ROS levels to survive, unraveling the interplay between oxidative stress and MB stem cells (MBSCs) metabolism could be crucial for MB therapy. It has been shown that radioresistant MBSCs display reduced levels of mitochondrial ROS and a marked reduction of oxidative phosphorylation compared to their non-stem counterparts (Sun et al., 2017).

It was recently hypothesized that a metabolic deviation from glycolysis to the PPP pathway underlies CSCs ability to face oxidative stress and avoid redox imbalance (Ghanbari Movahed et al., 2019). Oxidative PPP pathway essentially converts the glycolytic intermediate G6P into ribulose-5-phosphate (R5P) with the production of two NADPH molecules to protect CSCs from free radicals. These metabolic steps are achieved through the enzymatic activity of glucose-6-phosphate dehydrogenase (G6PD) and 6GPD. A comprehensive metabolic profiling of MB identified PPP signature as a prognostic factor for Group 3 MB (Park et al., 2019). Moreover, the authors found a strong association between two key PPP genes, Transketolase (TKT) and Transaldolase 1 (TALDO1), and poor prognosis in Group 3 and Group 4 MB patients (Park et al., 2019). This evidence suggests the importance of capitalizing the ROS-induced metabolic deviation to PPP pathway to trigger CSCs death. To this aim, inhibition of G6PD and 6GPD might be applied in combination with ROS-inducing therapies to target MB stem cells. Anti-PPP agents are still not available in clinical settings; however, several works pinpoint a potent and promising antitumor effect both *in vivo* and *in vitro* for different cancer types (Mele et al., 2018). Moreover, treatment with G6PD inhibitors could counteract therapy resistance, mainly driven by CSCs (Hong et al., 2018). Otherwise, a possible pitfall of this approach is the insurgence of a resistance phenotype due to the activation of metabolic ways alternative to PPP. This case has been recently explored in melanoma cells, where the impairment of G6PD led to increased malic enzyme activity and glutamine consumption to face oxidative stress (Aurora et al., 2022).

An emerging target for selectively killing CSCs with a PPP-dependent phenotype is the nuclear factor erythroid 2-related factor 2 (NRF2), the master regulator of the antioxidant defense, whose function in cancer biology has been extensively explored. NRF2 is a transcription factor that manages the overall cellular response and metabolic adaptation to oxidative stress by controlling the expression of many genes such as *G6PD* and *6PGD*. Under physiological conditions, the activity of NRF2 is inhibited by Kelch-Like ECH-Associated Protein 1 (Keap1), thus causing its cytoplasmic retention and its subsequent degradation. Upon oxidative stress, NRF2 dissociates from Keap1 and translocates into the nucleus where it trans-activates antioxidant responsive elements (ARE)-containing genes. A large body of evidence highlights a cytoprotective role for NRF2 in cancer progression and therapy resistance, including childhood brain tumors (Barrera et al., 2017; Godoy et al., 2020). Among neuroepithelial tumors, the World Health Organization (WHO) grade IV (the highest grade for CNS malignancies) MB shows the highest score of NRF2 staining (Barrera et al., 2017). In contrast to the significant amount of literature linking NRF2 with adult GBM, the current knowledge about its clinicopathological significance in MB is still poor. In a work from Tang et al., 2017 it has been demonstrated that NRF2 expression is enhanced in MB samples (n = 41) when compared to peritumoral control brain tissues (n = 27). Moreover, it was previously shown that pharmacological targeting of NRF2 with two NRF2-inducers (nifurtimox and tetrathiomolybdate) results in a synergic ROS-dependent antitumor response towards MB *in vitro* (Koto et al., 2011).

Otherwise, it is emerging that several anticancer drugs exert their cytotoxic function through the induction of intolerable levels of ROS. In line with this, it has been demonstrated that bortezomib, a proteasome inhibitor, which was previously found to selectively kill MB cells, induces cell death in a ROS-dependent manner (Ohshima-Hosoyama et al., 2011). Bortezomib treatment results in the stabilization of NOXA, a member of the Bcl-2 family, triggering apoptosis in response to high ROS levels in a p53-independent manner (Ohshima-Hosoyama et al., 2011; Hoerig et al., 2021). A recent molecular work from Li et al., has studied the effects of Liposomal Honokiol (Lip-HNK), a small bisphenol lignin delivered into liposomes, as a potential treatment against MB (Li et al., 2022). The authors found that HNK-dependent induction of apoptosis is mediated by ROS and thus reverted by treatment with the scavenger N-Acetylcysteine (NAC). In light of this data, it may be hypothesized that combinatorial approaches targeting both ROS production and specific metabolic adaptive mechanisms are needed to selectively kill MBSCs.

### Metabolic adaptation induced by cell-extrinsic factors

**FIGURE 3:**
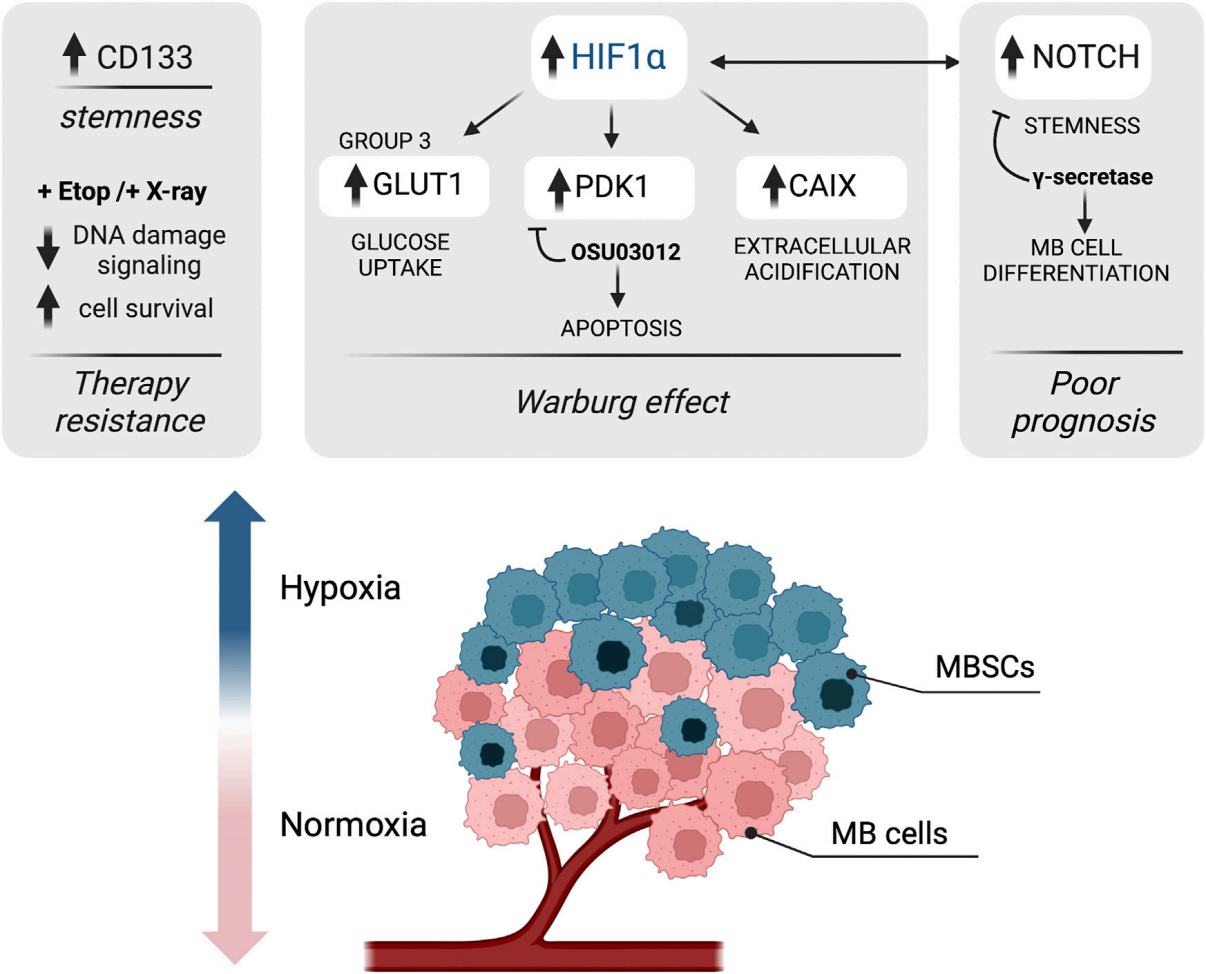
Schematic representation of the impact of hypoxia-associated metabolic adaptation in MB. Hypoxia is a major component of the tumor microenvironment that shapes tumor heterogeneity; the levels of oxygen in cancer cells decrease with increasing distance from the blood vessels. In hypoxic MB cells, hypoxia-inducible factor 1 alpha (HIF1α) supports the Warburg effect by upregulating the expression of genes involved in the glycolytic pathway, such as GLUT1, carbonic anhydrase IX (CAIX) and pyruvate dehydrogenase kinase 1 (PDK1). The use of PDK1 inhibitor OSU03012 induces mitochondrial-dependent apoptosis in MB. Moreover, hypoxic conditions were found to trigger upregulation of NOTCH, which correlates with a poor prognosis in MB. A connection between HIF1α and NOTCH signaling is also found, driving stemness and cancer stem cells (CSCs) expansion. Finally, hypoxia upregulates CD133 (staminal marker) expression in MB cells and contributes to therapy resistance (after both etoposide (Eto) and radiotherapy (X-ray) treatments) by attenuating DNA damage signaling. Figure is created in “BioRender.com”.

In addition to the complexity imposed by the oncogenotype and by cell-autonomous factors, environmental and cell-extrinsic factors are fundamental drivers of MB metabolism. During MB progression, cancer cells need to adapt their metabolism with respect to non-tumor cells to face the challenges posed by the harsh nature of the TME. Various metabolic stresses are encountered at different stages during MB development and relapse. TME is indeed generally hypoxic, acidic and with a distinct nutrient composition compared to non-tumor tissues, determining the first barrier that cancer cells must overcome in order to metastasize (Figure 3).

***The role of hypoxia in metabolic shaping of MB***. A common feature of solid tumors is the presence of regions with an imbalance in oxygen supply (Chouaib et al., 2016; Rankin and Giaccia, 2016). Cancer hypoxia is mainly caused by the uncontrolled proliferation of cancer cells that overcome the ability of the pre-existing blood vessels to satisfy the overall oxygen demand. Healthy cells are normally not viable at low concentrations of oxygen, which typically lead to cell death. On the contrary, cancer cells exploit genomic changes and modulate their entire metabolism to survive even at very low oxygen concentrations (pO_2_ ≤ 1%) (Al Tameemi et al., 2019) Hypoxia is clinically associated with a poor long-term prognosis and it is known to promote stemness and to block cell differentiation, thus favoring the selection and survival of CSCs within the TME. Due to the hard-to-practice techniques to measure pO_2_ in tumors and the lack of hypoxia-biomarkers, little is known about the extent of hypoxia in MB and other brain pediatric cancers.

Both in MB and glioma cell lines, it was demonstrated that *in vitro* induction of hypoxia increases the expression of the stem cell marker CD133 (Blazek et al., 2007). Moreover, low oxygen levels, particularly 2% oxygen, are required for *in vitro* expansion and survival of MB-derived precursor cells, whereas exposure to 20% oxygen induces tumor cell differentiation (Pistollato et al., 2010). Further, much evidence supports a role for hypoxia as an obstacle to therapy efficacy in brain tumors (Kitange et al., 2019; Mudassar et al., 2020). It was demonstrated that, after radiotherapy treatment under hypoxic conditions, MB cells display impaired DNA damage signaling by downregulating Nijmegen Breakage Syndrome 1 (NBS1) expression, thus increasing cell survival and limiting therapy effectiveness (Cowman et al., 2019). Understanding the mechanisms underlying MB adaptation to low oxygen is hugely important to develop and improve new targeted therapeutic strategies.

***HIF1α as a central regulator of hypoxia-induced metabolic adaptation of MB*.** At a molecular level, the adaptation of tumor cells to hypoxia is mainly orchestrated by a family of transcription factors known as Hypoxia Inducible Factors (HIFs), which are highly sensitive to decreased cellular oxygen levels and are able to regulate a plethora of cellular pathways. HIF1α, the main hypoxia effector, is upregulated in MB and its genetic downregulation has been associated with reduced MB cell proliferation (Cruzeiro et al., 2018). In several cancer types, the stabilization of HIF1α leads to the upregulation of glycolytic metabolic pathways to produce ATP, strengthening the typical cancer Warburg effect. Induction of HIF1α expression also enhances lactate production which leads to tumor hypoxic acidosis, as it was shown for glioma cells (Wang et al., 2017). All of these metabolic changes are associated with increased aggressiveness, radio and chemo-therapy resistance and poor prognosis in several tumors. Multiple therapeutic options to inhibit HIF1α-dependent signaling have been developed in the last years. Amongst them, one of the most promising approaches for MB could be targeting HIF1α downstream targets, as they are found to be frequently upregulated (see below). Here, we briefly review the potential of targeting upregulated HIF1α targets in MB (Figure 3).

***GLUT1*** *.* A downstream target of HIF1α that is known to contribute to hypoxia-induced metabolic adaptation of cancer cells is the pro-glycolytic factor GLUT1. HIF1α-dependent upregulation of *GLUT1* supports the maintenance of stem-like features as it was shown in GBM, where GLUT1 blockade has been shown to inhibit self-renewal of CSCs (Shibuya et al., 2015). Inhibitors targeting GLUT1, such as BAY-876, also restored radiosensitivity in resistant breast cancer cells (Zhao et al., 2016). Due to its important role in cancer-related metabolic adaptation, GLUT1 is a promising therapeutic target for glycolytic and hypoxic tumors like MB.

***PDK1***. Pyruvate dehydrogenase kinase 1 (PDK1) is another target gene that has been reported to be transcriptionally regulated by HIF1α. PDK1 has been defined as a ‘glycolysis gatekeeper’ due to its ability to promote glycolysis by blocking the entry of pyruvate into the TCA cycle. HIF1α-dependent induction of PDK1 is associated with glycolytic reprogramming, enhanced cancer cell proliferation and stemness in several tumors (Du et al., 2016; Semba et al., 2016; Peng et al., 2018). Therefore, genetically or chemically targeting of PDK1 would result in decreased energy availability, leading to cancer cell death. In 2010 Baryawno et al. demonstrated that PDK1 is constitutively activated in immunohistochemical sections of human primary MB samples. The authors have also shown that targeting PDK1 with the small-molecule inhibitor OSU03012 suppresses MB growth by inducing mitochondrial-dependent apoptosis both *in vitro* and *in vivo* (Baryawno et al., 2010). Moreover, PDK1 inhibition can potentiate the anti-tumor effect of mTOR inhibitor CCI-779 in *vivo* MB models (Baryawno et al., 2010). These results suggest that targeting the HIF1α-PDK1 axis could improve the therapeutic benefit in patients with hypoxic and metabolically-adapted MB.

***CAIX***. Hypoxia-induced changes in cellular metabolism include accumulation of lactate, as the end-product of glycolysis, together with carbon dioxide, which results in the acidification of the extracellular environment. Microenvironmental pH maintenance upon hypoxia in tumors relies on the functional activation of carbonic anhydrase IX (CAIX), a cancer-related transmembrane enzyme catalyzing the reversible conversion of carbon dioxide to bicarbonate ion and proton. Through its ability to regulate intracellular and extracellular pH, CAIX expression supports cancer cells survival in hypoxia/acidosis conditions and facilitates cancer cell migration and metastasization in several tumors (Becker, 2019; Hui et al., 2022). CAIX was found to be highly expressed in MB, especially in tumor areas with low microvascular density (Pistollato et al., 2010) and in perinecrotic areas (Nordfors et al., 2010). Importantly, CAIX expression significantly correlated with poor prognosis in a panel of MB patient samples (Nordfors et al., 2010). In recent years, inhibitors of CAIX such as SLC-0111, were shown to sensitize cancer cells to chemotherapy and are currently under evaluation for the treatment of metastatic solid tumors (McDonald et al., 2020). Given the role of CAIX expression in MB, it is plausible that this approach is also highly effective towards this pediatric cancer.

***NOTCH signaling and Hypoxia***. The interaction between HIF1α and NOTCH signaling plays an important role in promoting cell stemness (Gustafsson et al., 2005; Main et al., 2010). In normoxic conditions, NOTCH signaling is initiated when a cell-surface expressed ligand binds to the NOTCH receptor. This event triggers the cleavage of NOTCH intracellular domain (ICD) by ADAM metalloproteases and γ-secretase that translocates into the nucleus and induces the expression of target genes (Henrique and Schweisguth, 2019). Interestingly, HIF1α can directly interact with NOTCH ICD and allows its stabilization upon hypoxic conditions, sustaining NOTCH signaling in MB cells (Gustafsson et al., 2005; Pistollato et al., 2010). Inhibition of the NOTCH pathway by γ-secretase is sufficient to revert this effect, leading to MB cells differentiation upon hypoxia exposure (Pistollato et al., 2010). The implications of NOTCH signaling as an important driver of pluripotency and stemness in MB have been reported so far by several authors (Hallahan et al., 2004). NOTCH signaling pathway, indeed, promotes the development of both SHH and MYC-driven Group 3 MB, even though it is unable to initiate to MB in cerebellar precursors alone (Hallahan et al., 2004; Kahn et al., 2018; Ballabio et al., 2021). Additionally, SHH MB metastasization was found to be promoted by NOTCH pathway transcription factor Atonal Homolog 1 (ATOH1) (Grausam et al., 2017). Despite the role of NOTCH signaling in MB initiation, a recent analysis showed that NOTCH ligands and receptors are strongly associated with poor survival across MB patients, and NOTCH is most activated in both SHH and Group 3 samples (Northcott et al., 2017). Intriguingly, Mutvei and others found that HIF1α-dependent hyperactivation of NOTCH signaling induces a switch to HIF2α production in D341 cell line (MYC-amplified Group 3 MB) and not in DAOY cells (SHH MB) (Mutvei et al., 2018). Targeting the HIF1α-NOTCH axis could therefore affect hypoxia-driven oncogenic effects. Several NOTCH-targeting agents are now under clinical investigation for many cancer types and this approach might be beneficial especially for high-risk MB patients (Majumder et al., 2021).

**Nutrient availability within the TME**. Metabolic reprogramming arises as a consequence of the complex interplay between altered tumor properties and the tissue context that can fuel or hinder cancer proliferation. MB cells display an extensive crosstalk with both cellular (such as neurons, astrocytes and microglia) and non-cellular components of the brain extracellular matrix (ECM), which critically determine nutrient and growth factors supply. As reported above, it is widely accepted that MB can dramatically change its energy metabolism in response to nutrients availability in the TME. Although increasing attention has been focused on how environmental factors can shape MB evolution, the contribution of nutrient distribution to MB development and dissemination within primary and metastatic tumor sites remains yet unexplored. The case of pediatric brain tumors poses serious challenges due to the lack of experimental models that can faithfully reproduce the metabolic heterogeneity of both brain and leptomeningeal TME. In brain tumors, ECM is a main driver of metabolic adaptation; its composition and stiffness can determine the distribution of nutrients as well as the supply of oxygen to the tumor. Indeed, primary MB is surrounded by the ECM and mainly comes into contact with the interstitial matrix domain that is composed of loosely associated components (Van Ommeren et al., 2020). In contrast, leptomeningeal metastases are in contact with the basal lamina, which covers blood vessels and the pia mater and is mostly composed of a dense network of collagen, laminin and fibronectin. Therefore, in the leptomeningeal niche—the subarachnoid space—the concentration of growth factors and nutrients is dramatically different from the primary tumor (Van Ommeren et al., 2020). Moreover, during the metastasization process, MB cells must survive in a nutrient-deprived environment, especially when circulating through the CSF. With the exception of glutamine, CSF contains less glucose, amino acids and lipoproteins compared to blood. Recent studies are now focusing on the possible use of CSF as an indicator of MB disease status and also as a source of novel biomarkers. Metabolomic studies on MB CSF samples revealed high levels of metabolites that are typically secreted under hypoxia, such as α-KG, fumarate, hydroxypyruvate, malate and succinate as well as triacylglycerol (Lee et al., 2022). Moreover, recurrent MB patients-derived CSF samples show high concentrations of hypoxia-induced amino acids such as tryptophan, methionine, serine and lysine respectively (Reichl et al., 2020). These studies strengthen the concept that hypoxic and nutrient-deprived CSF represents a highly selective environment that may favor the adaptability of a small pool of metabolically flexible MB stem cells favoring tumor invasion and metastases.

***Interplay among nutrient availability and autophagy.*** The nutrient sensing pathways in the microenvironment are controlled by two master regulators, the mammalian target of Rapamycin complex 1 (mTORC1) and the AMP-activated Kinase (AMPK), which antagonize each other to balance cancer metabolic demands. AMPK and mTORC1 exert their function through the regulation of autophagy, a fundamental process that allows the recycling of intracellular components through their lysosomal-dependent degradation. Autophagy is a key survival process induced by nutrient stress and upregulated in several types of cancers, including high-risk MB (Nazio et al., 2019, 2021; Paul et al., 2020; Gatto et al., 2021). It has recently been demonstrated that autophagy activation in Group 3 MB supports stemness and metastasization (Nazio et al., 2021). Moreover, targeting autophagy through pharmacological inhibition with chloroquine (CQ) results in sensitization of Group 3 MB and significantly improves survival in orthotopic mouse models (Nazio et al., 2021). Conversely, mTOR is found upregulated in a high proportion of MB samples (Folgiero et al., 2016) and several works address mTOR targeting as a therapeutic strategy for MB (Dimitrova and Arcaro, 2015; Aldaregia et al., 2018; Eckerdt et al., 2019), even if the rise of treatment-resistant phenotype is a rather controversial issue (Alammar et al., 2021). mTOR activity is mainly regulated by nutrient levels and is particularly sensitive to amino acids, such as tryptophan, leucine and arginine (Saxton and Sabatini, 2017). In amino acids-deprived conditions, mTOR activity is inhibited, which promotes autophagy-dependent catabolism, providing energy and supporting cell survival. Radiotracing experiments demonstrated that tryptophan is greedily metabolized in MB patients (Xin et al., 2020) and its depletion from the tumor microenvironment is associated with the activity of tryptophan catabolic enzyme indoleamine 2,3-dioxygenase 1 (IDO1). mTORC1 inhibition by rapamycin treatment was able to increase IDO1 expression (Folgiero et al., 2016), suggesting the existence of a crosstalk between tryptophan levels and mTOR in MB, which is critical for the effectiveness of mTOR targeting approaches.

## Conclusion

**FIGURE 4:**
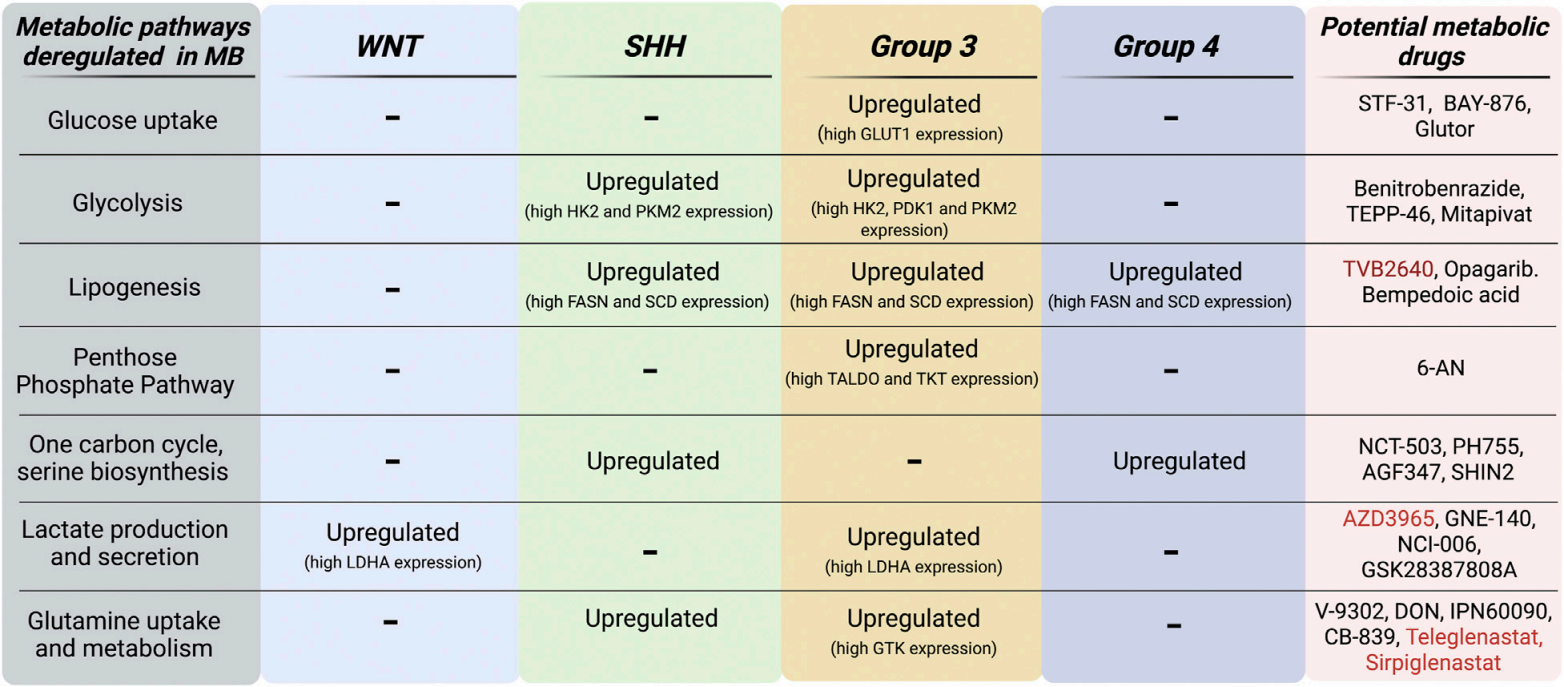
MB subgroup-specific alterations of metabolic pathways. Schematic representation of both metabolic pathways and genes that are found upregulated in MB subgroups. Metabolic inhibitors currently in preclinical (*black*) and clinical (*red*) development for oncology applications are listed in this scheme. Figure is created in “BioRender.com”.

Despite the lack of studies that compare tumor metabolism among MB subgroups, SHH MB tumors are the most well-characterized as Sonic Hedgehog, a mitogenic signal that directly regulates the bioenergetics of MB, drives metabolic dependence on aerobic glycolysis and lipogenesis rather on OXPHOS and fatty acids oxidation. However, low-carbohydrates ketogenic diets do not reduce tumor growth in allograft and spontaneous SHH-driven MB mouse models (Dang et al., 2015); thus, highlighting the presence of a complex scenario determining MB metabolic profiling and the possible pitfalls of therapeutic approaches targeting these changes. Indeed, human SHH MB tumors show high levels of OXPHOS and mitochondrial biogenesis (such as PGC1α) -associated genes (Łastowska et al., 2019), underlying the importance of mitochondrial-dependent metabolism that occur concomitantly to cytoplasmic metabolic reactions. It is not surprising that advanced solid tumors such as MB, after switching to glycolytic metabolism, remain dependent on mitochondria for key metabolic reactions such as glutaminolysis, fatty acid oxidation and for the synthesis of Krebs cycle intermediates that support tumor growth and spread. In light of this evidence, targeting the critical enzymes involved in metabolic adaptation to the microenvironment could be a valid approach to eradicate MB. However, progress in targeting cancer metabolism therapeutically in the past decade has been limited. Only a few metabolism-based drugs for cancer have been successfully developed, and some of them are currently being tested in clinical trials. Detailed descriptions about the currently available pharmacological compounds acting on metabolism together with the up-to-date clinical trials on tumors have been recently reviewed elsewhere (Lemberg et al., 2022; Stine et al., 2022). In this work, we have performed an unprecedented review of metabolism-related features of MB, detailing MB subgroup-specific metabolic alterations (Figure 4). Since intrinsical, genetic and clinical heterogeneity is a prominent feature of MB, it is of paramount importance to improve our knowledge about subgroup-specific molecular circuits adaptations to develop tailored target therapies for MB. Moreover, few metabolic drugs are already in clinical trials and it could be reasonable to promote repurposing of metabolism-based inhibitors for the treatment of MB. Here, we have summarized promising preclinical results of metabolic drugs tested directly against MB (Table 1). Importantly, even if the Warburg effect predominates MB metabolism, aerobic glycolysis and oxidative phosphorylation may coexist in MB and be differentially regulated throughout the malignant evolution of MB. However, the presence of intratumoral heterogeneity makes *in vitro* modeling of MB extremely difficult when studying metabolism. The use of 3D tumor organoids may reproduce MB heterogeneity but still lacks the high diversity of metabolites in the TME. Additionally, cell culture media contain nutrients such as glucose, amino acids and vitamins at levels that do not reflect those of human plasma or CSF and totally lack lipids and nucleotide precursors. Indeed, *in vivo* models better represent metabolic heterogeneity and TME, although mice diet, sex and environmental stressors are to be considered since they can have an impact on cancer metabolism. A very recent metabolomic analysis (Pham et al., 2022) shines a light on the discrepancies observed between *in vitro* MB cell cultures and orthotopic mouse xenografts in terms of metabolic phenotype, especially regarding glucose and glutamine uptake. This work showed how MYC-amplified orthotopic xenograft tumors display significantly higher glucose uptake and usage when compared to flank xenograft tumors and cells in culture (Pham et al., 2022). Together, this evidence suggests a crucial limitation of the metabolic profiling when conducted in non-native tumor environments. Beyond the experimental issue to *in vitro* model MB metabolism, comprehensive studies conducted both on patients and in mouse models have revealed a profound inter- and intra-variability based on the well-acknowledged subgroup MB classification. Therefore, staining for oncogenes and metabolic enzymes combined with assays measuring metabolic activities such as lactate production or glutamine uptake could help to stratify patients in order to develop personalized therapies. Moreover, the future entry of genomic, transcriptomic and metabolomic analyses into clinical routine will guide MB therapy integrating data considering both metabolic and genetic features.

**TABLE 1:** Preclinical studies targeting MB metabolism.

Metabolic pathway	Drug	Application/Mechanism of action	References
Glycolysis	Oxamate (pyruvate analog)	Proliferation and motility impairment in MB cell lines through LDHA inhibition and increased OXPHOS.	Valvona and Fillmore, (2018)
Glycolysis	GSK 2837808A (LDHA/B inhibitor)	Reduced MB cell invasion	Qin et al. (2022)
Glycolysis	OSU03012 (PDK1 inhibitor)	Induction of mitochondrial-dependent apoptosis *in vitro* and *in vivo* MB models	Baryawno et al. (2010)
Glycolysis	10058-F4 (MYC inhibitor)	Loss of SHH-induced HK2 upregulation in CGPCs by preventing MYC target genes expression	Gershon et al. (2013b)
Glutaminolysis	JHU395	Apoptosis induction in MYC- expressing MB cell lines; Increased survival in MYC-amplified MB mouse models	Hanaford et al., 2019
	JHU-083 (prodrugs of glutamine analog 6-diazo-5-oxo-l-norleucine)		Pham et al. (2021)
Glutamine transporter SLC1A5	None. Potentially druggable-target	non-WNT restricted MB.	Genovesi et al. (2021)
Inositol metabolism	Inositol hexakisphosphate (as single agent and combined with cisplatin)	Reduction of glycolysis and mTOR signaling; no OXPHOS activation in BMI1High; CHD7Low Group 4 MB cellular and *in vivo* models; enhanced IP6 cytotoxic effects in combination with cisplatin	Badodi et al. (2021)
Cholesterol biosynthesis	Simvastatin plus SMO antagonists (vismodegib or sonidegib)	Reduction of SHH MB cells proliferation and SHH-derived MB tumor growth in mice. Counteract SHH MB recurrence to SMO antagonist by inhibiting its cholesterol-mediated activation and SHH signaling	Gordon et al. (2018)
			Fan et al. (2021)
